# A Multi-Label Predictor for Identifying the Subcellular Locations of Singleplex and Multiplex Eukaryotic Proteins

**DOI:** 10.1371/journal.pone.0036317

**Published:** 2012-05-22

**Authors:** Xiao Wang, Guo-Zheng Li

**Affiliations:** The MOE Key Laboratory of Embedded System and Service Computing, Department of Control Science and Engineering, Tongji University, Shanghai, China; University of Alberta, Canada

## Abstract

Subcellular locations of proteins are important functional attributes. An effective and efficient subcellular localization predictor is necessary for rapidly and reliably annotating subcellular locations of proteins. Most of existing subcellular localization methods are only used to deal with single-location proteins. Actually, proteins may simultaneously exist at, or move between, two or more different subcellular locations. To better reflect characteristics of multiplex proteins, it is highly desired to develop new methods for dealing with them. In this paper, a new predictor, called **Euk-ECC-mPLoc**, by introducing a powerful multi-label learning approach which exploits correlations between subcellular locations and hybridizing gene ontology with dipeptide composition information, has been developed that can be used to deal with systems containing both singleplex and multiplex eukaryotic proteins. It can be utilized to identify eukaryotic proteins among the following 22 locations: (1) acrosome, (2) cell membrane, (3) cell wall, (4) centrosome, (5) chloroplast, (6) cyanelle, (7) cytoplasm, (8) cytoskeleton, (9) endoplasmic reticulum, (10) endosome, (11) extracellular, (12) Golgi apparatus, (13) hydrogenosome, (14) lysosome, (15) melanosome, (16) microsome, (17) mitochondrion, (18) nucleus, (19) peroxisome, (20) spindle pole body, (21) synapse, and (22) vacuole. Experimental results on a stringent benchmark dataset of eukaryotic proteins by jackknife cross validation test show that the average success rate and overall success rate obtained by **Euk-ECC-mPLoc** were 69.70% and 81.54%, respectively, indicating that our approach is quite promising. Particularly, the success rates achieved by **Euk-ECC-mPLoc** for small subsets were remarkably improved, indicating that it holds a high potential for simulating the development of the area. As a user-friendly web-server, **Euk-ECC-mPLoc** is freely accessible to the public at the website http://levis.tongji.edu.cn:8080/bioinfo/Euk-ECC-mPLoc/. We believe that **Euk-ECC-mPLoc** may become a useful high-throughput tool, or at least play a complementary role to the existing predictors in identifying subcellular locations of eukaryotic proteins.

## Introduction

Proteins perform their appropriate functions only when they are located in the correct subcellular locations. Therefore, one of the fundamental goals in cell biology and proteomics is to identify the subcellular locations of these proteins. Although the subcellular localization of a protein may be determined by carrying out various biochemical experiments, the approach by purely doing experiments is both time consuming and high cost. In the post-genomic age, the gap between newly found protein sequences and the information of their subcellular localization is becoming increasingly wide. To bridge such a gap, it is highly desirable to develop computational methods to predict protein subcellular localization automatically and accurately. During the past decade, many efforts have been devoted to deal with such a challenge, and a large number of computational methods have been developed in an attempt to predict the subcellular localization of proteins (see, e.g., [1]–[16] as well as a long list of references cited in two review papers [17], [18]).

Unfortunately, the aforementioned methods don't take multiple-location or multiplex proteins into account when predicting protein subcellular localization. In general, they were established under the assumption that a protein resides at one, and only one, subcellular location. However, proteins may simultaneously reside at, or move between, two or more different subcellular locations. Proteins with multiple location sites or dynamic feature of this kind are particularly interesting, because they may have some unique biological functions worthy of our special notice [19], [20]. In particular, recent evidences have indicated that an increasing number of proteins have multiple locations in the cell, as indicated by Millar et al. [21].

In this paper, we focus on predicting the subcellular locations of eukaryotic proteins with both singleplex and multiplex sites. So far, only three existing predictors, i.e., **Euk-mPLoc** [22], **Euk-mPLoc 2.0** [23] and **iLoc-Euk** [24], were developed that can be used to predict the subcellular locations of both singleplex and multiplex eukaryotic proteins. To the best of our knowledge, **iLoc-Euk** is at present the best predictor with capacity to deal with multiple-location or multiplex proteins when predicting eukaryotic protein subcellular localization. However, ML-KNN prediction engine used by **iLoc-Euk** is not optimal because it doesn't take correlations among subcellular locations into account.

In this paper, to better reflect the characteristics of multiplex proteins, a new predictor, called **Euk-ECC-mPLoc**, has been developed that can be used to deal with the systems containing both singleplex and multiplex eukaryotic proteins by introducing a powerful multi-label learning algorithm which exploits correlations between subcellular locations and by hybridizing the gene ontology information with the dipeptide composition information. Our experimental results on a benchmark dataset consisting of 7,766 eukaryotic protein sequences by jackknife cross validation test show that the overall success rates thus obtained by our proposed predictor **Euk-ECC-mPLoc** outperforms that by **iLoc-Euk** predictor. Moreover, for some subcellular locations with training proteins of very small size, the success rates achieved by **Euk-ECC-mPLoc** are 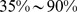 higher than those by **iLoc-Euk**. Therefore, **Euk-ECC-mPLoc** significantly improve the predictive performance on those “difficult” subcellular locations.

According to a recent comprehensive review [25], to establish a practically useful statistical predictor for a protein system, we need to consider the following procedures: (i) construct or select a valid benchmark dataset to train and test the predictor; (ii) formulate the protein samples with an effective mathematical expression that can truly reflect their intrinsic correlation with the target concerned; (iii) introduce or develop a powerful algorithm (or engine) to operate the prediction; (iv) properly perform cross-validation tests to objectively evaluate the anticipated accuracy of the predictor; (v) establish a user-friendly web-server for the predictor that is accessible to the public. Below, let us describe in detail how to deal with these steps one-by-one.

## Materials and Methods

### Dataset

In this paper, the dataset 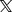 from **iLoc-Euk** [24] is used as the benchmark dataset for the current study. The dataset can be obtained from the Online Supporting Information S1 of [24]. The dataset is constructed specialized for eukaryotic proteins, where none of proteins included in 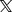 has greater than or equal to 25% pairwise sequence identity to any other one in a same subcellular location compared with most of the other benchmark datasets in this area. Using the dataset 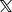 will make it more reliable and easier to compare our new predictor with the existing ones.

**Figure 1:**
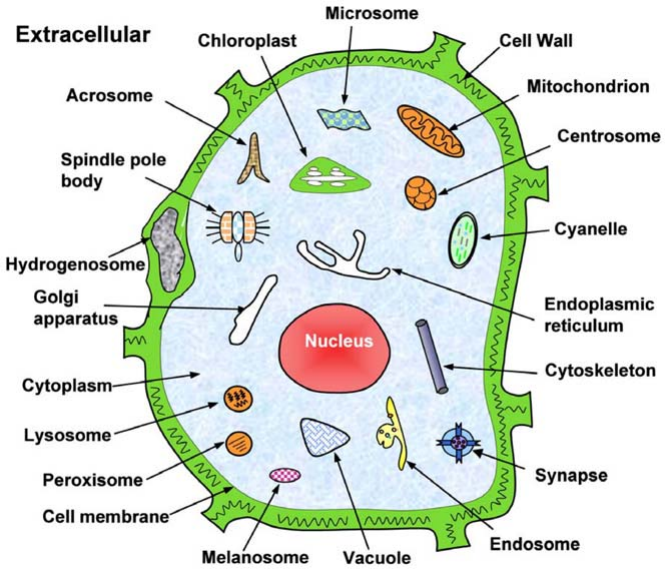
Schematic illustration to show the 22 subcellular locations of eukaryotic proteins. They are: (1) acrosome, (2) cell membrane, (3) cell wall, (4) centrosome, (5) chloroplast, (6) cyanelle, (7) cytoplasm, (8) cytoskeleton, (9) endoplasmic reticulum, (10) endosome, (11) extracellular, (12) Golgi apparatus, (13) hydrogenosome, (14) lysosome, (15) melanosome, (16) microsome (17) mitochondrion, (18) nucleus, (19) peroxisome, (20) spindle pole body, (21) synapse, and (22) vacuole. Adopted from [24] with permission.

The dataset 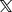 contains 7,766 different eukaryotic protein sequences, of which 6,687 belong to one subcellular location, 1,029 to two locations, 48 to three locations, and 2 to four locations. The dataset covers 22 different subcellular locations as shown in **Fig. 1**, and hence can be represented as
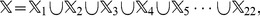
(1)where 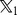 represents the subset for the subcellular location of “acrosome”, 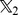 for “cell membrane”, 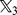 for “cell wall”, and so forth. A breakdown of the 7,766 eukaryotic proteins in the benchmark dataset 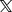 according to their 22 location sites is given in Table 1. To avoid redundancy and homology bias, none of the proteins in 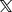 has greater than or equal to 25% pairwise sequence identity to any other in a same subset. For convenience, hereafter let us just use the subscripts of Eq.(1) as the codes of the 22 location sites; i.e., “1” for “acrosome”, “2” for “cell membrane”, “3” for “cell wall”, and so forth (Table 1).

**Table 1:** Breakdown of the eukaryotic protein benchmark dataset 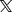 taken from [24].

Subset	Subcellular location	Number of proteins
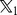	Acrosome	14
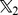	Cell membrane	697
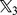	Cell wall	49
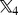	Centrosome	96
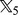	Chloroplast	385
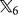	Cyanelle	79
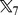	Cytoplasm	2186
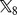	Cytoskeleton	139
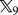	Endoplasmic reticulum	457
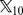	Endosome	41
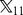	Extracellular	1048
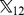	Golgi apparatus	254
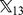	Hydrogenosome	10
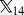	Lysosome	57
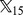	Melanosome	47
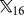	Microsome	13
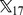	Mitochondrion	610
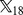	Nucleus	2320
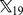	Peroxisome	110
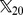	Spindle pole body	68
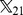	Synapse	47
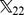	Vacuole	170
Total number of locative proteins N(loc)		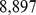
Total number of different proteins N(seq)		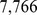

Note that because some proteins may occur in two different locations, the 7,766 different proteins actually correspond to 8,897 “locative proteins” (Table 1). For the concept of locative proteins, readers are referred to [22], [26], [27] where the difference between “protein” and “locative protein” and their relationship are elaborated.

### Feature Extraction

To develop a powerful method for statistically predicting protein subcellular localization, one of the most important steps is to extract core and essential features of protein samples that are closely correlated with their subcellular locations. To avoid losing important information hidden in protein sequences, the pseudo amino acid composition (PseAAC) was proposed [28], [29] to replace the simple amino acid composition (AAC) for representing the sample of a protein. For a brief introduction about Chou's PseAAC, please visit the Wikipedia web-page at http://en.wikipedia.org/wiki/Pseudo_amino_acid_composition. For a summary about its recent developments and applications, see a comprehensive review [30]. Ever since the concept of PseAAC was proposed by Chou [28] in 2001, it has rapidly penetrated into almost all the fields of protein attribute prediction, such as identifying bacterial virulent proteins [31], predicting homo-oligomeric proteins [32], predicting protein secondary structure content [33], predicting supersecondary structure [34], predicting protein structural classes [35], [36], predicting protein quaternary structure [37], predicting enzyme family and sub-family classes [38]–[40], predicting protein subcellular location [41]–[44], predicting subcellular localization of apoptosis proteins [45]–[48], predicting protein subnuclear location [49], predicting protein submitochondria locations [50]–[52], identifying cell wall lytic enzymes [53], identifying risk type of human papillomaviruses [54], identifying DNA-binding proteins [55], predicting G-Protein-Coupled Receptor Classes [56], [57], predicting protein folding rates [58], predicting outer membrane proteins [59], predicting cyclin proteins [60], predicting GABA(A) receptor proteins [61], identifying bacterial secreted proteins [62], identifying the cofactors of oxidoreductases [63], identifying lipase types [64], identifying protease family [65], predicting Golgi protein types [66], classifying amino acids [67], among many others. Actually, according to a recent comprehensive review [25], the Chou's PseAAC is generally formulated as
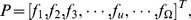
(2)where the subscript 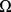 is an integer, and its value as well as the components depends on how to extract the desired features from the amino acid sequence of P.

In the present study, we adopt *Gene Ontology* and *Dipeptide Composition* feature extraction methods to generate features of protein examples, which are widely used in many existing protein subcellular localization systems [22]–[24], [26], [27], [68]–[74]. For reader's convenience, a brief introduction on *Gene Ontology* and *Dipeptide Composition* is given below.

#### Gene Ontology

*GO* database [75] was established according to the molecular function, biological process, and cellular component. The following questions might be raised by those who do not really understand GO (Gene Ontology): One of the three aspects of GO is ‘Cellular Compartment’ [75], which is just another name for subcellular location. If a protein already has GO annotation, why does one need to predict its subcellular location? Is it merely a procedure of converting the annotation into another format? Is it true that the high success rate obtained via the GO approach was due to a trivial utilization of the subcellular component annotations in the GO database? To really understand these questions, the readers should carefully read the paper [14], particularly the profound and penetrating analysis on the left column of page 155 of that paper [14]. For readers' convenience, it can be briefly summarized as follows: (i) Although GO database is constructed based on protein function and cellular component, for those proteins with ‘subcellular location unknown’ annotation in Swiss-Prot database, most (more than 99%) of their corresponding GO numbers in GO database are also annotated with ‘cellular component unknown’. (ii) Even for those proteins whose subcellular locations are clearly annotated in Swiss-Prot database, their corresponding GO numbers in GO database do not always directly indicate their corresponding subcellular locations. In some cases they are actually annotated with ‘cellular component unknown’. (iii) More important, it should be emphasized that during the course of prediction, only the GO numbers of a query protein but not its GO annotations were used, just like the case of using all the other predictors in identifying the protein subcellular location that only the sequence of a query protein but not its Swiss-Prot annotation was used. (iv) Finally, as shown by the compelling statistical analysis given in Table 6 of the paper [14], the percentage (45.02%) of proteins with GO annotations to indicate their subcellular components is even less than the percentage (51.76%) of proteins with known subcellular location annotation in the Swiss-Prot database. Accordingly, the high success rate obtained by the method via the GO approach was by no means due to a trivial procedure of converting the annotation from one into another format, as often misinterpreted by some people. Furthermore, it can be seen from Table 6 of the paper [14] that there is a huge number of proteins with given accession numbers and the corresponding GO numbers, but their subcellular locations are still unknown. Actually, the essence of why using GO approach to represent protein samples can significantly improve the prediction quality is due to the fact that proteins mapped into the GO database space would be clustered in a way better reflecting their subcellular locations, thus to significantly enhances the success rate of prediction for those proteins that do not have significant sequence homology to proteins with known locations, as elaborated in [18], [76]. So far, there are two main approaches to extract features from *GO* database space. However, in order to incorporate more information, instead of only using 0 and 1 elements as done in [23], here let us use another better approach [24] as described below.

#### Step 1

Compression and reorganization of the existing *GO* numbers. The *GO* database (version 94 released on 08 April 2011) contains many *GO* numbers. However, these numbers do not increase successively and orderly. For easier handling, some reorganization and compression procedures are taken to renumber them. The *GO* database obtained through such a treatment is called GO_compress database, which contains 18,844 numbers increasing successively from 1 to the last one.

#### Step 2

Using Eq.(2) with 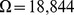, the protein P is represented as
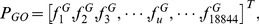
(3)where 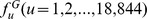 are defined via the following steps.

#### Step 3

Use BLAST [77] to search the homologous proteins of the protein P from the Swiss-Prot database (version 55.3), with the expect value 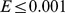 as the BLAST parameter.

#### Step 4

Those proteins which have 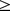
60% pairwise sequence identity with the protein P are collected into a set, 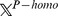, called the “homology set” of P. All the elements in 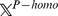 are deemed as the “representative proteins” of P, sharing some similar attributes such as structural conformations and biological functions [78]–[80]. Because they were retrieved from the Swiss-Prot database, these representative proteins must have their own accession numbers.

#### Step 5

Search the *GO* database at http://www.ebi.ac.uk/GOA/ to find the corresponding *GO* number(s) [81] for each of the accession numbers collected in Step 4, and then convert the *GO* numbers thus obtained to their GO_compress numbers as described in Step 1. (Note that the relationships between the UniProtKB/Swiss-Port protein entries and the *GO* numbers may be one-to-many, “reflecting the biological reality that a particular protein may function in several processes, contain domains that carry out diverse molecular functions, and participate in multiple alternative interactions with other proteins, organelles or locations in the cell” [75]. For example, the Uni-ProtKB/Swiss-Prot protein entry “P01040” corresponds to three *GO* numbers, i.e., “GO:0004866”, “GO:0004869”, and “GO:0005622”).

#### Step 6

The elements in Eq.(3) is given by
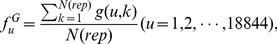
(4)where 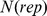 is the number of representative proteins in 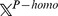, and
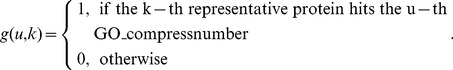
(5)


Note that the *GO* feature extraction method may become a naught vector or meaningless under any of the following situations: (1) the protein P does not have significant homology to any protein in the Swiss-Prot database, i.e., 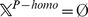 meaning the homology set 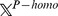 is an empty one; (2) its representative proteins do not contain any useful *GO* information for statistical prediction based on a given training dataset.

Under such a situation, let us consider using the dipeptide composition method as backup to extract features for the protein P, as described below.

#### Dipeptide Composition

Dipeptide composition (abbreviated as DC) represents the co-occurrence frequency of each two adjacent amino acid residues. It is used to describe the global information about each protein sequence in the form of 420-dimensional (420-D) feature vector. An advantage of *DC* over amino acid composition is that it uses some sequence-order information. Dipeptide composition generates 420 components for each protein sequence, the first 20 components are the conventional amino acid composition(AAC); the following 400 components are the fractions of 400 dipeptides, i.e. AA, AC, AD, … , YV, YW, YY; the 400 components are calculated using the following equation
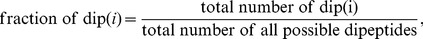
(6)where dip(i) is the i-th dipeptide of the 400 dipeptides, i = 1, 2 ,…, 400.

### Prediction Algorithm: Ensemble of Classifier Chains

To enhance the success rate, the powerful ECC (Ensemble of Classifier Chains) classifier [82] is adopted to perform prediction. Below, let us introduce the Ensemble of Classifier Chains classifier.

Without lose of generality, let us consider a system or dataset 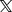 that contains 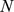 eukaryotic proteins classified into 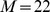 subcellular location sites. The dataset 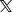 can be represented by the following matrix:
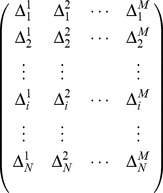
(7)where 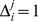 (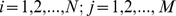) if the 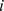-th eukaryotic protein belongs to the 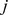-th subcellular location site, 0 otherwise.

According to Eq.(7), we know that if 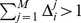, the 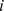-th eukaryotic protein is a multiplex protein, while if 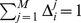, the 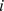-th eukaryotic protein is a single-location protein. In this study, we deal with the case that there is at least one eukaryotic protein of 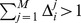, that is to say, the systems that contain both single-location and multiple-location eukaryotic proteins.

Before introducing Ensemble of Classifier Chains, we firstly present a simple method, called Binary relevance (BR) [83], which converts a multi-label learning problem into a number of independent binary classification ones. Taking the above system or dataset 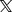 for example, 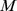 independent binary classifiers are separately constructed for the 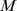 eukaryotic subcellular location sites, i.e.,
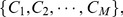
(8)where 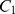 is the prediction model for the 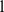
st subcellular location site, 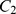 for 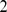
nd and so on. The positive (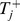) and negative (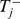) training samples for 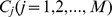 are collected according to the following formula:
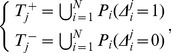
(9)where 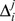 represents the label information as shown in Eq.(7), 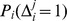 represents the protein that belongs to the 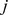-th subcellular location site, 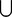 is the union symbol in the set theory.

**Figure 2:**
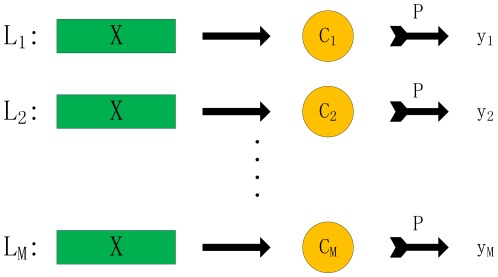
Figure to illustrate the complete process of BR method.

For the prediction of a query protein, BR outputs the union of the class labels that are predicted by the 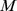 classifiers:
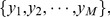
(10)where 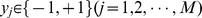 is the result predicted by the 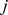-th classifier, 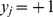 representing the query protein belonging to the 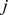-th subcellular location site, otherwise not. To provide an intuitive picture, it is shown in **Fig. 2** to illustrate the complete process of BR method.

BR is conceptually simple and easy to implement, whereas may be less effective since it don't take label correlations into account. In the experiment below, we will compare our proposed ECC method with the BR method in order for proving the effectiveness of considering label correlations.

**Figure 3:**
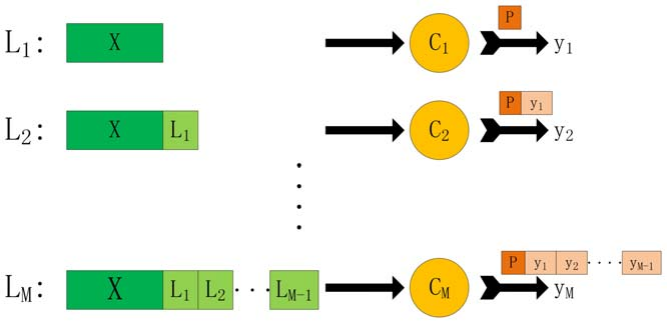
Figure to illustrate the complete process of ECC method.

Now we begin to introduce ECC algorithm. ECC algorithm is proposed by J.Read in [82], which aggregates multiple CC (Classifier Chain). CC is the core of the ECC algorithm, which is based on the framework of BR and consists of 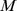 classifiers as in BR. However, in contrast to BR, classifiers are linked along a chain where each classifier is responsible for prediction of presence or absence of one class label. The feature space of each classifier in the chain is extended with the 0/1 class label associations of all previous classifiers. In other words, assuming that the classifier chain 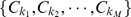 (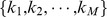 is a random permutation of 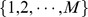) is constructed, each classifier 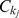 in the chain is responsible for predicting the binary association of class label 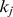 given the feature space, augmented by all prior binary relevance associations in the chain 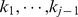. An intuitive illustration is provided in **Fig. 3**.

The chaining method passes label information between classifiers, allowing CC to take into account label correlations and thus overcoming the label independence problem of BR method. However, the order of the chain itself clearly has an effect on accuracy. In [82], the issue is solved by using an ensemble framework with a random chain ordering for each iteration.

In contrast to the traditional single-label ensemble learning, ECC is an ensemble of multiple multi-label methods, i.e. the CC method. Following the typical strategy of ensemble learning, ECC also has two steps, in which the first is to train 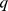 CC classifiers 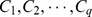 and the second is to combine their predictions. In the first step, each 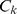 is trained with both a random chain ordering and a random subset of original training data set. In the second step, multi-label predictions of each 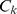 classifier are summed by label so that each label gets some votes, and then, we use a threshold to select the most possible labels which form the final multi-label prediction. Specifically, each 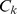 classifier predicts a vector 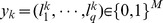. The sums are stored in a vector 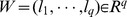 such that 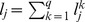. Hence each 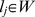 represents the sum of the votes for the 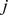
th label. We then normalize 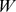 to 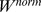, which represents a distribution of scores for each label in [0, 1]. A threshold is used to choose the final multi-label set 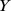 such that class label 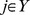 if 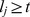 for threshold 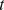. Here we simply set the threshold to be 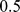. Hence the relevant labels in 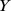 represent the final multi-label prediction.

Support vector machine (SVM) [84] is a powerful binary classifier in the field of machine learning and pattern recognition. The basic ideas behind SVM is to map the input vectors into a high dimensional feature space and then find an Optimal Separating Hyperplane (OSH) which maximizes the margin, i.e., the distances between the hyperplane and the nearest data points of each class in the mapped feature space. SVM classifier has been largely and successfully used in the field of prediction of protein subcellular localization [3]–[5], [8]–[11]. In this study, we also use Support vector machine (SVM) as base classifier in both BR and ECC. The software package used to train SVM with default parameters is the very efficient LIBLINEAR library [85] which is specially designed for large scale and high dimensional datasets as the benchmark eukaryotic protein dataset for the current study.

**Figure 4:**
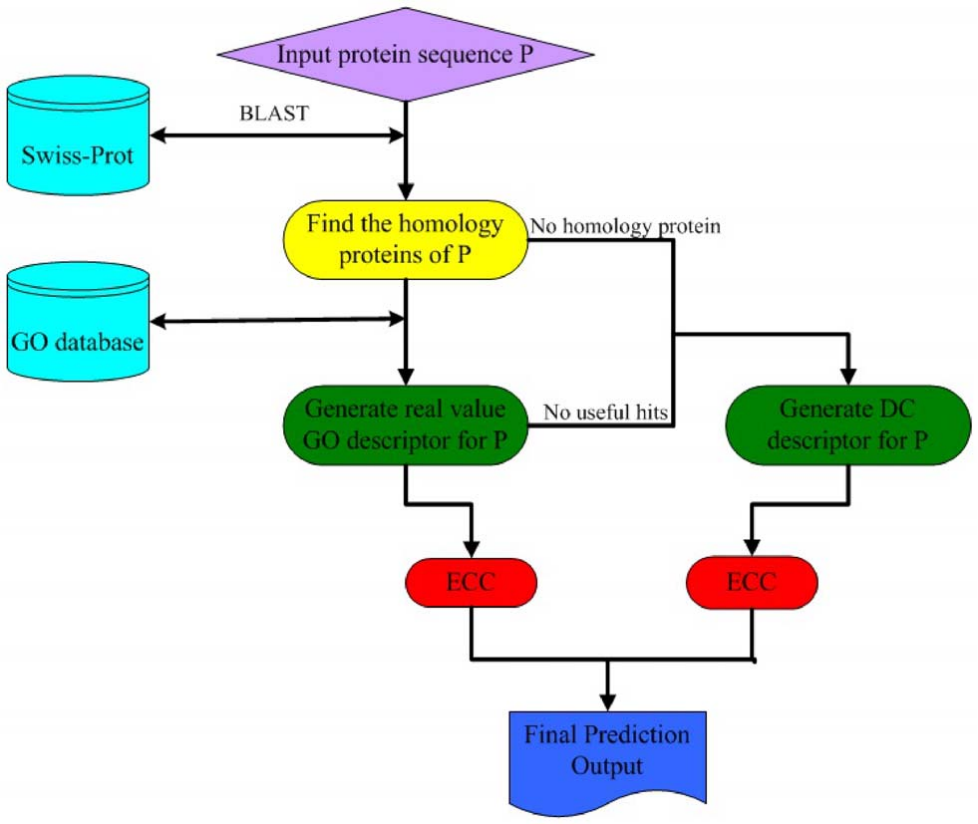
A flowchart to show the prediction process of Euk-ECC-mPLoc.

The entire predictor thus established is called **Euk-ECC-mPLoc**, which can predict the subcellular localization of both singleplex and multiplex eukaryotic proteins. To provide an intuitive picture, a flowchart is provided in **Fig. 4** to illustrate the prediction process of **Euk-ECC-mPLoc**.

## Results and Discussion

In statistical prediction, it is needed to evaluate the quality of different prediction methods. The following three commonly used methods, that is, the independent data set test, K-fold cross validation test, and jackknife test, are often used for evaluating the power of a statistical prediction method. Of the three methods, the jackknife test is deemed as the most objective because it always generates a unique result for a given benchmark dataset, as elucidated in a comprehensive review [18]. Therefore, the jackknife test has been increasingly and widely employed by researchers to examine the accuracy of various prediction methods (see, e.g., [23], [24], [26], [86]–[88]). Accordingly, in the present study, we use jackknife test to evaluate the power of **Euk-ECC-mPLoc**.

Actually, for such a stringent and complicated dataset containing both single-location and multiple-location eukaryotic proteins distributed among 22 subcellular location sites, so far only three existing predictors, i.e., **Euk-mPLoc** [22], **Euk-mPLoc 2.0** [23] and **iLoc-Euk** [24], were able to deal with it. It has been reported from [23] that, **Euk-mPLoc 2.0**, which is an updated version of **Euk-mPLoc**, can significantly outperform **Euk-mPLoc**. Moreover, as can be seen from [24], the overall jackknife success rate achieved by **iLoc-Euk** was about 15% higher than that by **Euk-mPLoc 2.0** when tested on the dataset 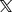. As a result, **iLoc-Euk** is currently the best one. Therefore, to demonstrate the power of the proposed predictor, it would suffice to just compare **Euk-ECC-mPLoc** with **iLoc-Euk**.

Table 2 reports the detailed results on the 22 eukaryotic subcellular locations obtained with **iLoc-Euk** and **Euk-ECC-mPLoc** on the aforementioned benchmark dataset 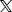 by the jackknife test. For a fair algorithmic comparison between **Euk-ECC-mPLoc** and **iLoc-Euk**, we use the same GOA database version that is described in this study to extract *GO* features for **Euk-ECC-mPLoc** and **iLoc-Euk**. As can be seen from Table 2, for such a stringent and complicated dataset, the average jackknife success rate achieved by **Euk-ECC-mPLoc** is 69.70%, which is about 19% higher than that achieved by **iLoc-Euk** [24]. **Euk-ECC-mPLoc** achieves very satisfactory performance on most subcellular locations, whereas **iLoc-Euk** achieves very poor performance on some subcellular locations, e.g., “acrosome”, “endosome”, “hydrogenosome”, “melanosome” and “microsome”. It is indicated that **Euk-ECC-mPLoc** is more balanced than **iLoc-Euk**. Meanwhile, **Euk-ECC-mPLoc** obtains 81.54% overall jackknife success rate, with about 3% performance improvement against **iLoc-Euk**. For the benchmark dataset containing both singleplex and multiplex eukaryotic proteins, the prediction accuracy is mainly influenced by the multiplex characteristics of proteins in that location. Roughly speaking, the bigger multiplex protein ratio in a location, the lower success rate will be obtained. For example, there are about 32% and 60% proteins respectively in the “melanosome” and “synapse” location belonging to two or more locations, **iLoc-Euk** obtains only 2.13% and 38.30% success rates respectively. **Euk-ECC-mPLoc**, however, achieves 53.19% and 46.81% success rates in the two locations respectively, with largely 51% improvement in the “melanosome” location and over 8% improvement in the “synapse” location. The main reason is that correlations between different subcellular location sites have been taken into account in our proposed **Euk-ECC-mPLoc**, while **iLoc-Euk** only transforms the problem of predicting multiplex eukaryotic protein subcellular locations to a number of problems of prediction of singleplex eukaryotic protein subcellular localization, and thus **iLoc-Euk** lose much important information related to multi-label learning problems, e.g., correlations between different subcellular locations as utilized in **Euk-ECC-mPLoc**. Therefore, **Euk-ECC-mPLoc** reaches better performance than **iLoc-Euk** in predicting multiplex proteins. Moreover, for some subcellular locations with smaller number of training proteins, the success rates achieved by **Euk-ECC-mPLoc** are 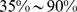 higher than those by **iLoc-Euk**. For example, the success rate by **Euk-ECC-mPLoc** in “hydrogenosome” is 90% higher than that by **iLoc-Euk**, and the success rate by **Euk-ECC-mPLoc** in “acrosome” is about 64% higher than that by **iLoc-Euk**. This may be caused by the inherent advantage of SVM base classifier used in **Euk-ECC-mPLoc**.

**Table 2:** A comparison of the jackknife success rates by iLoc-Euk [24] and the proposed Euk-ECC-mPLoc on the benchmark dataset 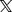 that covers 22 location sites of eukaryotic proteins in which none of the proteins included has 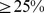 pairwise sequence identity to any other in a same location.

Code	Subcellular location	Success rate by jackknife test	
		iLoc-Euk	Euk-ECC-mPLoc
1	Acrosome	7.14%	71.43%
2	Cell membrane	80.49%	79.20%
3	Cell wall	16.33%	51.02%
4	Centrosome	69.79%	66.67%
5	Chloroplast	87.79%	87.01%
6	Cyanelle	64.56%	60.76%
7	Cytoplasm	76.72%	77.77%
8	Cytoskeleton	27.34%	28.78%
9	Endoplasmic reticulum	89.06%	87.96%
10	Endosome	7.32%	36.59%
11	Extracellular	90.46%	91.60%
12	Golgi apparatus	63.39%	69.29%
13	Hydrogenosome	0.00%	90.00%
14	Lysosome	31.58%	73.68%
15	Melanosome	2.13%	53.19%
16	Microsome	0.00%	38.46%
17	Mitochondrion	77.05%	83.11%
18	Nucleus	87.93%	87.28%
19	Peroxisome	54.55%	85.45%
20	Spindle pole body	66.18%	83.82%
21	Synapse	38.30%	46.81%
22	Vacuole	71.76%	83.53%
Average		50.45%	69.70%
Overall		79.06%	81.54%

Table 3 illustrates the “exact match” success rate between predicted outputs and real annotations on the same benchmark dataset 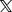 by the jackknife test. The “exact match” means that both the predicted number and annotations of the subcellular locations for a query protein are the same as real observations. For a protein belonging to three subcellular locations, if only two of the three are correctly predicted, or the predicted result contains a location not belonging to the three, the prediction score will be counted as 0. In other words, when and only when all the subcellular locations of a query protein are exactly predicted without any underprediction or overprediction, can the prediction be scored with 1. Meanwhile, the success rates by the random predictor are also shown. Because **iLoc-Euk** didn't provide the accuracy value specific to each subset in terms of the number of subcellular locations, the corresponding values are set to be “-”. As can be seen from Table 3, the overall “exact match” success rate achieved by **Euk-ECC-mPLoc** is 72.59%, which is slightly higher than 71.27%, the corresponding “exact match” success rate achieved by **iLoc-Euk** [24]. The “exact match” accuracy of **Euk-ECC-mPLoc** is significantly superior to the random predictor. Therefore, our approach is quite promising for handling multiplex proteins, or at least play a complementary role to the existing predictors in identifying the subcellular locations of eukaryotic proteins.

**Table 3:** A comparison of the jackknife “exact match” success rates by iLoc-Euk [24] and the proposed Euk-ECC-mPLoc on the benchmark dataset 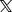 that covers 22 location sites of eukaryotic proteins in which none of the proteins included has 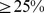 pairwise sequence identity to any other in a same location.

Number of Locations	Euk-ECC-mPLoc	iLoc-Euk	Random
1	75%	-	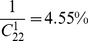
2	59.09%	-	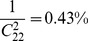
3	10.42%	-	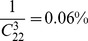
4	0%	-	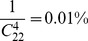
Overall	72.59%	71.27%	-

In order to make the readers understand the superiority of our approach than other existing predictors more easily and intuitively, several typical proteins that are localized in multiple subcellular locations are selected from DBMLoc [89] which is a database of proteins with multiple subcellular localizations, and thus make prediction by inputting them into our **Euk-ECC-mPLoc** and **iLoc-Euk** online web servers respectively. Results are listed in Table 4 with the predicted outputs by the two predictors and the corresponding experimental annotations. As can be seen from Table 4, predicted subcellular locations achieved by our approach are all identical to the corresponding true annotations, whereas **iLoc-Euk** fails to get fully accurate results.

**Table 4:** the predicted outputs by iLoc-Euk and Euk-ECC-mPLoc as well as the corresponding experimental annotations from DBMLoc [89].

UniProt entry	UniProt entry name	Locations predicted by iLoc-Euk	Locations predicted by Euk-ECC-mPLoc	Annotations in DBMLoc
P38143	GPX2_YEAST	Cytoplasm	Cytoplasm	Cytoplasm
			Nucleus	Nucleus
P25823	TUD_DROME	Mitochondrion	Cytoplasm	Cytoplasm
			Mitochondrion	Mitochondrion
P28829	BYR2_SCHPO	Cytoplasm	Cell membrane	Cell membrane
			Cytoplasm	Cytoplasm
P32614	FRDS_YEAST	Cytoplasm	Cytoplasm	Cytoplasm
		Mitochondrion	Mitochondrion	Mitochondrion
		Nucleus		
Q9H190	SDCB2_HUMAN	Cytoplasm	Cell membrane	Cell membrane
			Cytoplasm	Cytoplasm
Q9Y7Q2	GST1_SCHPO	Cytoplasm	Cytoplasm	Cytoplasm
			Nucleus	Nucleus
O59827	GST2_SCHPO	Cytoplasm	Cytoplasm	Cytoplasm
			Nucleus	Nucleus
P27476	NSR1_YEAST	Nucleus	Mitochondrion	Mitochondrion
			Nucleus	Nucleus
P47119	ITPA_YEAST	Nucleus	Cytoplasm	Cytoplasm
			Nucleus	Nucleus

### Conclusion

Prediction of protein subcellular localization is a challenging problem, particularly when the system concerned contains both singleplex and multiplex proteins. In this paper, we have proposed a novel multi-label predictor, called **Euk-ECC-mPLoc**, for predicting eukaryotic protein subcellular locations based on the powerful ECC algorithm and a hybrid of GO and DC feature extraction methods, which has been demonstrated powerful for dealing with both singleplex and multiplex proteins. Since user-friendly and publicly accessible web-servers represent the future direction for developing practically more useful predictors [90], here we have provided a web-server for the method presented in this paper at http://levis.tongji.edu.cn:8080/bioinfo/Euk-ECC-mPLoc/. The current approach represents a new strategy to deal with the multi-label biological problems, and hence may become a useful tool in the areas of bioinformatics and proteomics.
